# Gene Expression in Peripheral Immune Cells following Cardioembolic Stroke Is Sexually Dimorphic

**DOI:** 10.1371/journal.pone.0102550

**Published:** 2014-07-18

**Authors:** Boryana Stamova, Glen C. Jickling, Bradley P. Ander, Xinhua Zhan, DaZhi Liu, Renee Turner, Carolyn Ho, Jane C. Khoury, Cheryl Bushnell, Arthur Pancioli, Edward C. Jauch, Joseph P. Broderick, Frank R. Sharp

**Affiliations:** 1 Department of Neurology and MIND Institute, University of California Davis, Sacramento, California, United States of America; 2 Cincinnati Children's Hospital Medical Center, University of Cincinnati, Cincinnati, Ohio, United States of America; 3 Department of Neurology, Wake Forest University Medical Center, Winston-Salem, North Carolina, United States of America; 4 Department of Emergency Medicine, University of Cincinnati, Cincinnati, Ohio, United States of America; 5 Division of Emergency Medicine, Medical University of South Carolina, Charleston, South Carolina, United States of America; 6 University of Cincinnati Neuroscience Institute, Department of Neurology, Cincinnati, Ohio, United States of America; University of Jaén, Spain

## Abstract

**Aims:**

Epidemiological studies suggest that sex has a role in the pathogenesis of cardioembolic stroke. Since stroke is a vascular disease, identifying sexually dimorphic gene expression changes in blood leukocytes can inform on sex-specific risk factors, response and outcome biology. We aimed to examine the sexually dimorphic immune response following cardioembolic stroke by studying the differential gene expression in peripheral white blood cells.

**Methods and Results:**

Blood samples from patients with cardioembolic stroke were obtained at ≤3 hours (prior to treatment), 5 hours and 24 hours (after treatment) after stroke onset (n = 23; 69 samples) and compared with vascular risk factor controls without symptomatic vascular diseases (n = 23, 23 samples) (ANCOVA, false discovery rate p≤0.05, |fold change| ≥1.2). mRNA levels were measured on whole-genome Affymetrix microarrays. There were more up-regulated than down-regulated genes in both sexes, and females had more differentially expressed genes than males following cardioembolic stroke. Female gene expression was associated with cell death and survival, cell-cell signaling and inflammation. Male gene expression was associated with cellular assembly, organization and compromise. Immune response pathways were over represented at ≤3, 5 and 24 h after stroke in female subjects but only at 24 h in males. Neutrophil-specific genes were differentially expressed at 3, 5 and 24 h in females but only at 5 h and 24 h in males.

**Conclusions:**

There are sexually dimorphic immune cell expression profiles following cardioembolic stroke. Future studies are needed to confirm the findings using qRT-PCR in an independent cohort, to determine how they relate to risk and outcome, and to compare to other causes of ischemic stroke.

## Introduction

Experimental, clinical and epidemiological evidence points to sexual dimorphism in stroke for: risk factors, age, heritability, causes, outcomes and response to treatment [1], [2] For example, females have more cardioembolic stroke, while men have more large-vessel and lacunar stroke [3], [4]. The heritability of stroke is higher in females than males [2]. Sex differences in response to thrombolysis and functional outcome have also been suggested [5], [6]. Hormonal as well as non-hormonal mechanisms have been proposed for these sex differences [1], [7], [8].

We have previously investigated the sexually dimorphic immune response in blood of patients with acute ischemic stroke [9]–[13]. The current study investigates the sex differences in gene expression in the peripheral immune system following cardioembolic stroke, a major cause of stroke. We hypothesized that some of the sex differences in cardioembolic stroke relate in part to differences of cell death, coagulation and inflammatory pathways in peripheral leukocytes. We found more regulated genes in females than in males and found differences in time course of expression of neutrophil–specific genes in females compared to males following cardioembolic stroke.

## Materials and Methods

Study protocols were approved by the institutional review boards (IRB) at each site (University of California at Davis and University of Cincinnati) and written informed consent was obtained from each patient. Detailed methods, including diagnosis of cardioembolic stroke and analysis details, are provided in File S1. In brief, whole blood from subjects with cardioembolic stroke was collected at three time points following stroke (n = 23, 11 female, 12 male; 69 samples). Treatment [14] was initiated within 3 hours of onset in all studied stroke patients. The first blood sample was drawn before treatment (tPA treatment alone, or in combination with eptifibatide therapy) (≤3 hours). After treatment, two blood samples were drawn at 5 hours and 24 hours after the stroke. The vascular risk factor controls (VRFC) (n = 23; 11 female, 12 male) were subjects with no history of symptomatic vascular disease [15].

Gene expression was analyzed on Affymetrix U133 Plus 2.0 whole-genome expression arrays as a function of diagnosis, age, race, sex, batch and sex-by-diagnosis interaction using ANCOVA. Genes with false discovery rate (FDR)-corrected P≤0.05 (multiple comparison correction) and |fold change| ≥1.2 were considered differentially expressed. An additional ANOVA model accounted for vascular risk factors (VRFs  =  atrial fibrillation, diabetes, hyperlipidemia, hypertension). Genes significant for any VRF (p<0.005) were excluded. For the analysis, females with cardioembolic stroke were compared with female vascular risk factor controls (VRFC), and males with cardioembolic stroke were compared with male VRFC. Thus female stroke and control subjects were compared to each other, and male stroke and control subjects were compared to each other to minimize the effect of genes with sex-associated expression. A Fisher's Exact test, a Chi-square test and a t-test were performed for the demographic and clinical characteristics, and a p<0.05 was considered a significant difference. The data from this study have been deposited in GEO, accession number GSE58294.

## Results

### Subject Demographics

Subjects' characteristics are presented in Table 1. Age was not statistically significantly different between male and female strokes, nor between male and female controls (VRFC). Age was significant between stroke and VRFC males (*P* = 1.2E-05), and between stroke and VRFC females (*P* = 7E-04). Race was not significant between stroke and VRFC males, stroke and VRFC females, and female and male VRFCs. Race was significant between female and male stroke subjects (*P* = 9.4E-03). No VRFs are significantly different between female and male stroke subjects, and between female and male VRFCs. Across the groups, the only significant differences were female stroke vs VRFC for atrial fibrillation (p = 1.2E-02), and male stroke vs VRFC for hyperlipidemia (p = 3.9E-02). As noted above, we controlled for the differences in VRF by including them in the ANCOVA model, and by excluding genes significant for any of the VRFs. NIH Stroke Scale (NIHSS) was not significantly different between female and male stroke patients (Table 1).

**Table 1:** Subject characteristics.

	Vascular Risk Factor Controls			Cardioembolic Stroke Patients		
	Total	Male	Female	Total	Male	Female
Subjects, no.	23	12	11	23	12	11
Age, years (mean±SD)	57.9±3.3	56.8±3.6	59.1±2.6	71.7±7.9	72.1±7.6	71.3±8.5
Q1	55.0	53.8	56.5	69.1	71.0	63.4
Median	58.0	56.0	61.0	74.9	74.2	68.1
Q3	61.0	60.3	61.0	77.5	77.0	77.3
Race						
White, no. (%)	19 (82.6)	11 (91.7)	8 (72.7)	15 (65.2)	11 (91.7)	4 (36.4)
Nonwhite, no.(%)	4 (17.4)	1 (8.3)	3 (27.3)	8 (34.8)	1 (8.3)	7 (63.6)
Hyperlipidemia, no. (%)	16 (69.6)	9 (75)	7 (63.6)	6 (26.1)	3 (25.0)	3 (27.3)
Hypertension, no. (%)	16 (69.6)	9 (75)	7 (63.6)	16 (69.6)	8 (66.7)	8 (72.7)
Diabetes, no. (%)	5 (21.7)	3 (25.0)	2 (18.2)	4 (17.4)	2 (16.7)	2 (18.2)
Atrial fibrillation, no. (%)	0 (0)	0 (0)	0 (0)	9 (39.1)	3 (25.0)	6 (54.5)
NIHSS, <3 h	N/A	N/A	N/A	15.4	15.0	15.9
Q1				10.0	8.3	11.5
Median				13.0	13.5	13.0
Q3				20.0	20.3	18.5
NIHSS, 5 h	N/A	N/A	N/A	11.9	11.7	12.0
Q1				5.8	6.5	6.5
Median				9.5	10.0	9.0
Q3				16.0	14.0	15.0
NIHSS, 24 h	N/A	N/A	N/A	11.2	10.2	12.3
Q1				3.5	3.0	4.5
Median				10.0	10.0	12.0
Q3				16.0	13.5	18.0

### Sexual dimorphism <3 h following cardioembolic stroke (prior to treatment)

**Figure 1:**
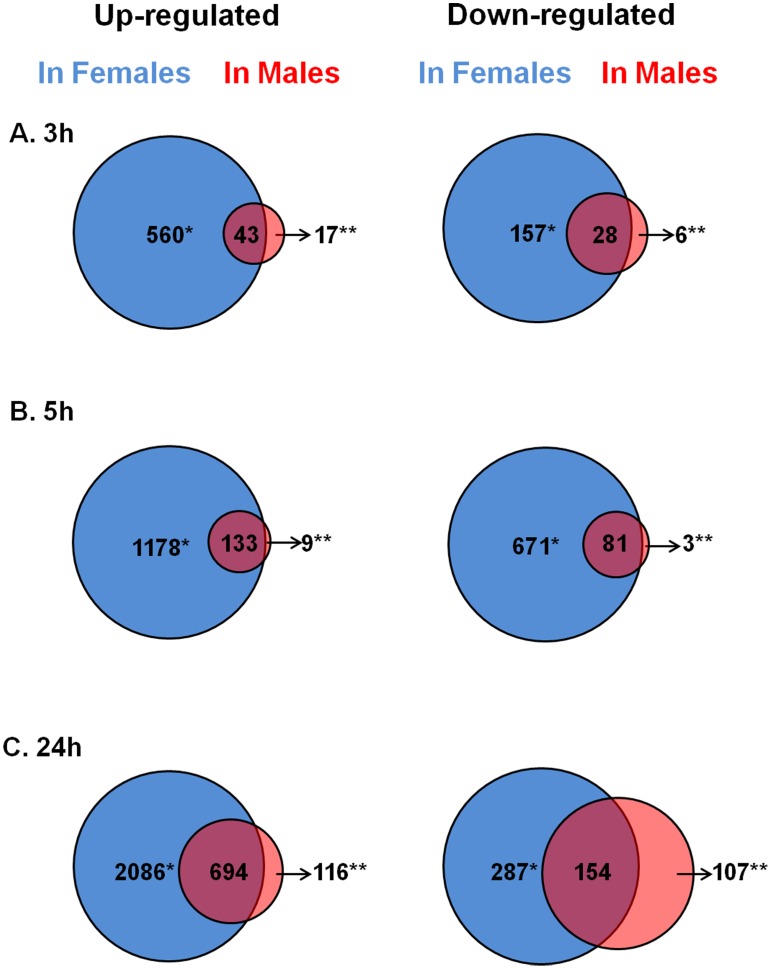
Genes differentially expressed in female and male following cardioembolic stroke. * -genes with female-specific expression; ** -genes with male-specific expression.

The results and discussion focus on this time point because subjects received no treatment prior to this blood draw. A total of 788 genes (1,118 probe sets) were differentially expressed in females with stroke compared to VRFC females, and 94 genes (140 probe sets) were differentially expressed in males with stroke compared to VRFC males. Of the 788 and 94 genes, 717 were female-specific, 23 were male-specific, and 71 genes were common to both sexes (Table S1 in File S2). Generally, many more genes were up regulated rather than down regulated in both sexes; and more genes were regulated in females compared to males (Figure 1A).

There were 14 over-represented canonical pathways (Benjamini-Hochberg FDR corrected P<0.05) in females (in the 788 female genes) (Table S2 in File S2), the top five of which are presented in Table 2. No canonical pathway at ≤3 h in males (in the 94 male genes) passed the FDR corrected P-value threshold.

**Table 2:** Top five over-represented canonical pathways in cardioembolic stroke; Benjamini-Hochberg P-value <0.05.

Females, ≤3 h following cardioembolic stroke	Females, 5 h following cardioembolic stroke
1. Toll-like Receptor Signaling	1. Toll-like Receptor Signaling
2. PPARα/RXRα Activation	2. B cell Receptor Signaling
3. Hypoxia Signaling in the Cardiovascular System	3. Role of JAK Family Kinases in IL-6-type Cytokine Signaling
4. TREM1 Signaling	4. EGF Signaling
5. IL-10 Signaling	5. IL-22 Signaling

Females, 24 h following cardioembolic stroke	Males, 24 h following cardioembolic stroke
1. B Cell Receptor Signaling	1. B Cell Receptor Signaling
2. Toll-like Receptor Signaling	2. Toll-like Receptor Signaling
3. NF-kB Signaling	3. Fcγ Receptor-mediated Phagocytosis in Macrophages and Monocytes
4. EIF2 Signaling	4. T Cell Receptor Signaling
5. Fcγ Receptor-mediated Phagocytosis in Macrophages and Monocytes	5. FGF Signaling

The highest scoring Network in females was Cell Death, Survival, Assembly and Organization, which was similar to males with Cellular Assembly, Organization and Compromise (Figure S1 in File S1). The top Disease and Functions annotation in females was inflammatory response, including several neutrophil processes, such as influx, recruitment, cell movement, and homing; phagocytosis of blood cells, chemotaxis of phagocytes, and phagocytosis and chemotaxis of myeloid cells. These processes were predicted to be significantly increased (P<3.9×10^−03^; activation z-score>2) according to Ingenuity Pathway Analysis (IPA) knowledge-based predictions (see Table S3 in File S2). See Supplementary Material for how Z score and statistical significance were calculated. The Disease and Functions IPA annotations with the highest activation z-score for the 94 differentially expressed genes in males was Development of Blood Vessels and Vasculogenesis (P = 0.03; z-score = 1.8) (see Table S3 in File S2).

**Figure 2:**
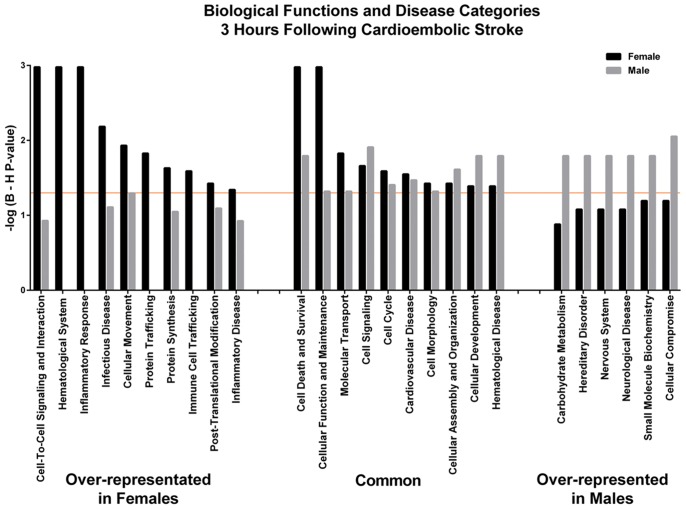
Over-represented categories in the female-specific and/or male-specific genes regulated at ≤3 hours following cardioembolic stroke. Categories above the orange line are significant at Benjamini-Hochberg corrected P-value <0.05.

Based on the expression of the genes in males, the transcription factor XBP1 was predicted to be activated (P = 0.0003, activation z-score = 2.2), while in females predicted transcriptional regulators included XBP1, NFKBIA, KLF5, EIF4E and HIF1A (hypoxia inducible factor 1, alpha subunit) (P = 0.01; activation z-score>2) (see Table S4 in File S2). Xbp1 is involved in the unfolded protein response to cell stress, NFKBIA regulates oxidative stress responsive genes, EIF4E regulates translation, and HIF1A regulates hypoxia responsive target genes. Genes differentially expressed only in male or only in female cardioembolic strokes are presented in Table S1 in File S2. Figure 2 represents over-represented Functions and Disease Categories in these female- and male-specific gene lists at ≤3 hours following cardioembolic stroke. There are several Functions common to both sexes (Figure 2).

### Sexual dimorphism at 5 h following cardioembolic stroke

A total of 1,972 genes (2,937 probe sets) were differentially expressed in females with stroke compared to VRFC females, and 226 genes (304 probe sets) were differentially expressed in males with stroke compared to VRFC males (see Table S1 in File S2). Of these genes, 1,758 were female-specific, 12 were male-specific, and 214 were common to both sexes (see Table S1 in File S2). Figure 1B describes the numbers of up-regulated and down-regulated stroke genes compared to the VRFC. The over-represented canonical pathways (Benjamini-Hochberg P<0.05) in female are presented in Table 2 (top 5) and Table S2 in File S2. No canonical pathway at 5 h in males passed the FDR corrected P-value threshold.

The highest scoring network in females was Cell-to-Cell Signaling and Interaction and Inflammatory Response, while in males it was Post-Translational Modification, DNA Replication, Recombination, and Repair. The top Disease and Functions annotations for the 1,972 differentially expressed genes in females (activation z-scores>2) were infectious disease, protein transport, leukocyte development, polarization of macrophages, T cell development/homeostasis, RNA induction, polarization of antigen presenting cells, engulfment by macrophages, and response/ influx of macrophages; whereas in males it was proliferation of cells and transcription (see Table S3 in File S2). Genes differentially expressed only in male or only in female cardioembolic stroke subjects at 5 h are presented in Table S1 in File S2.

### Sexual dimorphism at 24 h following cardioembolic stroke

At 24 hours post-stroke, 3,213 genes (5,161 probe sets) were differentially expressed in females with stroke compared to VRFC females, and 1,070 genes (1,474 probe sets) were differentially expressed in males with stroke compared to VRFC males (see Table S1 in File S2). Of these genes, 2,362 were female-specific, 219 were male-specific, and 851 were common to both sexes (see Table S1 in File S2). The over-represented canonical pathways at 24 h in females and males are presented in Table 2 (top 5) and Table S2 in File S2.

The highest scoring network in females was post-translational modification, humoral immune response and protein synthesis; while in males it was RNA post-transcriptional modification, cardiovascular disease and cell cycle. The top Disease and Functions in females were cell death and survival, response to phagocytes, engulfment by macrophages, chemotaxis of neutrophils, recruitment and response of phagocytes, differentiation of antigen presenting cells, T cell development and homeostasis and cardiovascular system function processes; while in males it was chemotaxis, homing and cytotoxicity of leukocytes, and quantity of T lymphocytes (see Table S3 in File S2). Genes differentially expressed only in male or only in female cardioembolic stroke subjects at 24 h are presented in Table S1 File S2.

### Sexually dimorphic cell-specific response to cardioembolic stroke

**Figure 3:**
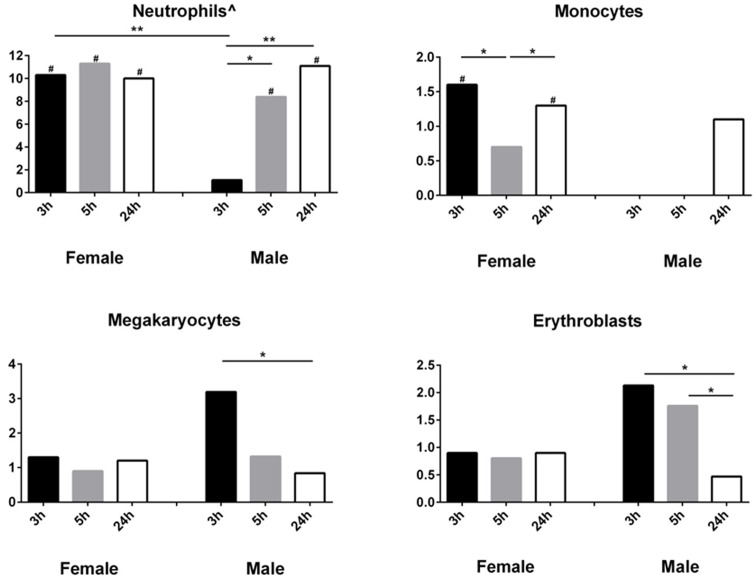
Cell-type specific gene expression in female and male following cardioembolic stroke. Y-axis - % of genes from our gene list, which overlap with a cell-type specific gene list from Watkins et al, 2007. * p<0.05; **P<0.005; # hypergeometric probability of overlap between our gene list and the cell-type specific gene list from Watkins et al, 2007<0.05; ∧ Watkins et al used positive selection, therefore granulocyte population consisted of three cell types (neutrophils, eosinophils, and basophils), all of which expressed CD66b and would have therefore been co-purified. However, the neutrophils are the largest percent.

We next investigated transcripts expressed in just one blood cell type. To do this we compared our gene lists with the Haem Atlas gene lists [16]. We calculated the percentages of regulated cell-specific transcripts (transcripts uniquely expressed in only one cell type) in our gene lists and plotted them on the Y axis in Figure 3. The percentage was calculated as the number of transcripts differentially expressed at each time point for each sex (our gene lists) that are known to be specific for each cell type (based on overlap with the Haem Atlas cell-specific transcripts) divided by the total number of transcripts in each of our gene lists. Since the numbers of lineage-specific genes vary greatly for each cell type (752 unique for neutrophils, 190 for monocytes, 262 for megakaryocytes, 210 for B cells, 303 for erythroblasts, 44 for helper T cells and 74 for Natural Killer cells) [16], the percent of transcripts from our gene lists that overlap with the cell-type specific transcripts is greatly influenced by these numbers. A t-test for significant differences between percents was used to test for significant differences between the sexes and times. The data show the percents of neutrophil-specific transcripts were over-represented in the gene lists at ≤3, 5 and 24 h in females but only at 5 h and 24 h in males (Figure 3). The data also show significant percentages of monocyte-specific transcripts in females at ≤3 h and 24 h, and decreases in percentages of megakaryocyte and erythrocyte genes from ≤3 h to 24 h in males (Figure 3). The changes for the other cell types were not statistically significant (Figure S2 in File S1). Since we measured gene expression in whole blood (containing different cell types), the only way to deduce cell-type specific expression for this sub-analysis is by looking at transcripts unique to a particular blood cell type, based on the HaemAtlas transcript catalogue. Thus, as an example, the lack of monocyte-specific genes differentially expressed at ≤3 h in males does not mean that monocytes in males did not have differentially expressed genes at this time point – it just means there were no “monocyte-specific” genes.

## Discussion

Gene expression following cardioembolic stroke showed common and sex-specific changes. There were more up-regulated than down-regulated genes in both sexes and more genes regulated in females than in males, in line with previous studies [11], [12]. The findings point to sexual dimorphism of cell death, cell survival, inflammation, and coagulation pathways in peripheral blood and support our previous findings of an important role for neutrophils in cardioembolic stroke [9], [10], [17], which we show here is sexually dimorphic.

### Early immune response following cardioembolic stroke (≤3 h, untreated)

#### Female response

A number of interconnected pathways (Figure S3 in File S1) previously associated with ischemic stroke were over-represented in females, including TLR (Toll-like receptor), HMGB1, TREM1, PPARα/RXRα, Hypoxia, Renin-Angiotensin, Mitochondrial Dysfunction, Glucocorticoid receptor and cytokine signaling pathways [12], [18]. TLR Signaling was the top over-represented pathway at all three time points in females (but only at 24 h in males). It plays a pivotal role in stroke and preconditioning and serves as a link between the central nervous system and periphery after stroke. TLR2,5,8, MYD88, and a number of TLR modulators – IRAK1, MEKK1, NIK, p38MAPK, NF-kB, MKK3/6 were up regulated in females, most having been implicated in ischemic brain injury [19]. Toll-like receptors have a number of downstream effects following cerebral ischemia, including MyD88-directed activation of neutrophil chemoattractants [20], and this process was predicted to be increased in females in this study. In addition, HMGB1 (high-mobility group protein B1), released by injured endothelial and other cells following ischemic stroke, activates TLR2/4 which leads to ROS generation and cytokine production [21].

#### Male response

The highest scoring network in males at 3 h had convergent nodes on CREB, Caspase-1, and ERK1/2, all having been implicated in stroke (Figure S3 in File S1). Cerebral ischemia triggers phosphorylation of CREB and subsequent CRE-mediated gene expression in neurons, which leads to expression of genes encoding neuroprotective molecules [22]. CASP1 is involved in forming the inflammasome and initiating inflammation via activation of IL-1β. A number of transcriptional regulators belonged to this network including YY1, SMARCA4, HNRNPD, TRIM27 and ATRX. The YY1 transcription factor modifies histones by directing histone deacetylases and acetyltransferases to activate or repress promoter sites. SMARCA4 (SWI/SNF related, matrix associated, actin dependent regulator of chromatin, subfamily a, member 4) is part of the SNF/SWI complex and regulates transcription by altering the chromatin structure around specific genes. HNRNPD (heterogeneous nuclear ribonucleoprotein D (AU-rich element RNA binding protein 1, 37 kDa) regulates mRNA stability. These findings may suggest that different epigenetic mechanisms are involved in the early response to stroke in males, one possible explanation for the smaller number of differentially expressed genes in males compared to females. These differences in the immune response by 3 hours after stroke in males and females suggest differences in clotting and/or the immune response to the ischemic brain injury and may contribute to the sexually dimorphic features of cardioembolic stroke [23].

### Cell Death Pathways in Peripheral Blood Following Cardioembolic Stroke

Brain cell death and apoptosis pathways are sexually dimorphic in experimental stroke models [24], with females often showing caspase-mediated cell death of neurons whereas males predominantly undergo caspase-independent cell death [8]. Notably, in our previous study of human peripheral blood leukocytes following ischemic stroke, we also observed sexually dimorphic cell death pathways with females showing Caspase 1, HMGB1, and NFkB Signaling pathways, while in males Granzyme A signaling and other cell death molecules were involved [12]. Similarly in the current study at ≤3 hours following cardioembolic stroke, we observed 217 molecules involved in cell death in females, such as CASP1 (caspase1), IRAK3, CREB1, HMGB1, CARD11, NFkB1, BCL2A1, ITGAM (integrin, alpha M), TLR2, INRAF1 (interferon (α, β, γ) receptor 1), STAT5B (signal transducer and activator of transcription 5), CD44, CD59, and MYD88. ITGAM is important in adherence of neutrophils and monocytes to stimulated endothelium, and also in the phagocytosis of complement coated particles. ITGAM regulates ICAM1, Fibrinogen, TNF, IL6, ITGB2, NFkB, IL1B, CYR61, STAT3, MAPK3, MAPK1, IFNB1, C3 and superoxide - all of which have been implicated in ischemic brain injury [7]. These molecules belonged to TLR receptor, HMGB1, NFkB, TREM1 and other signaling pathways (Table S2 in File S2). In addition, Apaf-1 (apoptotic associated factor 1), FADD (Fas (TNFRSF6)-associated via death domain) and Bcl-2, up regulated in female cardioembolic stroke, have all been associated with female cell death following stroke [7].

In contrast, 22 molecules were involved in cell death in males at ≤3 hours, including NOA1 (nitric oxide associated 1), TRAF7 (TNF receptor-associated factor 7, E3 ubiquitin protein ligase), and several transcriptional regulators (ATRX, SMARCA4, TFDP1, TRIM27, YY1). NOA1 is involved in regulation of mitochondrial protein translation and respiration. It plays a role in mitochondria-mediated cell death. TRAF7 is a signal transducer for members of the TNF receptor superfamily, which are death receptors in the extrinsic apoptosis pathway. Notably, 18 out the 22 male cell death genes at ≤3 hours were among the 217 female cell death genes. These and previous results [7], [8], [25] suggest common and divergent pathways of cell death in males and females. Though the cell death genes discovered here relate to peripheral blood leukocytes and other cells, they are important since they are being expressed in peripheral blood cells that interact with and/or enter brain, and might relate to endogenous immune responses of brain in cells like microglia which are very much like blood monocytes.

### Coagulation System

Clotting is important in the pathogenesis of human strokes. Transcripts of many coagulation factors and platelet activating genes increased in both sexes, though most with different temporal profiles. F5 (coagulation factor V) was increased at all three time points in females, while only at 24 h in males. F12 (coagulation factor XII, Hageman factor), which increased both in males and females at the later time points, initiates the intrinsic pathway of coagulation and together with the extrinsic pathway forms fibrin. F5 has been consistently implicated in stroke, while F12's involvement has recently been elucidated [26]. Another gene involved in the clotting cascade, GP1BB (glycoprotein Ib (platelet), beta polypeptide) was up regulated only in females at 24 h following cardioembolic stroke. GP1BB is part of the GPIb-V-IX system that constitutes the receptor for von Willebrand factor (VWF) and mediates platelet adhesion in the arterial circulation.

PAFAH1B1 (platelet-activating factor acetylhydrolase 1b, regulatory subunit 1 (45 kDa)) and PAFAH1B2 (platelet-activating factor acetylhydrolase 1b, catalytic subunit 2 (30 kDa)) also show sexually dimorphic expression being regulated at all three time points in females but only at later time points in male cardioembolic strokes. This sex difference in the coagulation system suggests it can have implications for risk factors, progression of ischemic injury, outcome and response to treatment. Treatment with tPA causes fibrinolysis of blood clots and improves outcomes. However, sexual dimorphism in response to tPA treatment has been suggested [5], [6]. Thus, since the clot is an important clinical target, consideration for a sexually dimorphic coagulation system should be considered in animal and clinical studies in stroke.

### Female-specific Inflammatory Response

Inflammatory response pathways were associated with female-specific genes at ≤3 hours after cardioembolic stroke (Figure 2). This included predicted increase in several biological processes, such as recruitment, homing, chemotaxis and influx of neutrophils, immune response by phagocytes, immune response by antigen presenting cells and antimicrobial and antiviral response. Inflammation and infection are linked to stroke risk and outcome, with some evidence for sexual dimorphism (reviewed in [27]–[29]). The immune system can contribute to risk of stroke and to damage and/or to repair processes following stroke (reviewed in [27]–[29]). These sex differences could also contribute to some of the sexual dimorphic features of stroke, such as age at stroke, response to treatment and outcome [30]. In addition, both inflammatory and anti-inflammatory pathways are enriched in female at <3 h following stroke. Though other possible explanations exist, it is plausible that one reason for this complex response is different cell types in the blood are mounting different immune responses.

### Sexual Dimorphism in Cell-Type Specific Gene Expression

The cell-specific transcriptional changes of immune cells in blood following cardioembolic stroke differed in males and females, with neutrophil-specific genes being the most predominantly expressed genes at ≤3 h, 5 h and 24 h in females, while males showed changes at 5 h that increased at 24 h. These findings support our previous findings where genes regulated in cardioembolic stroke were found to be associated more with neutrophils, while the ones regulated in large-vessel atherosclerotic stroke were associated more with platelets and Monocytes [10].

Neutrophils, the most abundant cell type in human blood, are involved in response to infectious stimuli and non-infectious inflammation [19]. They migrate to the site of inflammation or injury where they remove dead cells and release inflammatory molecules [19]. In stroke they have recently been shown to have important roles in clotting and atherosclerosis [31]. Moreover, increased numbers of neutrophils in blood have been correlated with the risk of having an ischemic stroke and myocardial infarction as well as correlating with poor outcomes and increased mortality [32]. The greater percentage of neutrophil-specific transcripts in females compared to males at early times following stroke in this study could relate to biological functions associated with the increased rate and morbidity of cardioembolic stroke in females [30].

Of interest the percentage of megakaryocyte-specific transcripts was highest at ≤3 h and decreased at later time points in males, with no such temporal effects being observed in females. Megakaryocytes are responsible for producing platelets, which are needed for the formation of a blood clot. Sexually dimorphic megakaryocyte responses may be associated with the suggested differential benefit of anti-platelet therapy in males and females [5].

## Limitations

It is important to emphasize that though this is the first description of gene expression changes in blood related to sex in cardioembolic stroke, it is a pilot study with small sample size. Future studies are needed to validate the findings using qRT-PCR in an independent cohort. qRT-PCR was not performed here since this is an initial study, but once the genes are biologically validated in an independent cohort then they will be technically validated using qRT-PCR in a future study. Some of the sexually dimorphic gene expression differences may reflect pre-existing differences not accounted for in our analyses. There were more female than male stroke subjects with atrial fibrillation, and none of the controls had asymptomatic atrial fibrillation; thus some of the results could be due to sexual dimorphism related to atrial fibrillation. In addition, differences in smoking, BMI, medications, and other possible confounders could not be accounted for because of the small number of subjects in this pilot study. Thus some of the sexually dimorphic gene expression changes could be due to these pre-existing baseline differences and not be associated with the stroke response. The VRFC control subjects were younger than the stroke subjects. We tried to account for this by including age in the ANCOVA model, and by performing the analyses across groups (Stroke vs. VRFC) for each sex, thus having similar age-related bias in the male and female analyses. Nevertheless, some of the observed differences could be age-related. Since the 5 h and 24 h time points were collected after treatment, and because we do not yet have untreated cardioembolic stroke samples for these time points, we cannot estimate how much of the dimorphic response is due to a sexually dimorphic response to treatment at these time points [5], [25]. Thus, the discussion focused on the genes and pathways affected before treatment at ≤3 hours. Since cardioembolic strokes were not compared to other causes of stroke because of insufficient numbers of subjects (males and females), future studies will be needed to determine which of the sexually dimorphic changes identified here are specific for cardioembolic stroke. Since this study only examined gene expression changes between 3 and 24 h following stroke, no expression data was available beyond 24 hours that might also affect outcomes in men versus women following cardioembolic stroke. Differences of hormone status of stroke compared to control subjects were controlled for in part by comparing female stroke to female controls and male stroke to male controls. However, there are complex hormonal differences between females with stroke compared to males with stroke that likely affect gene expression and that relate in part to the post-menopausal status of most of the women and declining testosterone levels in older men. Finally, this is an observational study and though some of the differences may relate to different hormonal status in males versus females, other causes for the differences remain to be determined.

## Conclusions

More genes are up regulated than down regulated following cardioembolic stroke and more genes are regulated in females than males. Sexually dimorphic gene expresison is noted for genes in cell death and coagulation pathways and for genes specifically expressed by neutrophils, monocytes and megakaryocytes following cardioembolic stroke. This study provides additional evidence for the sexually dimorphic nature of cardioembolic stroke.

## Supporting Information

File S1 **Includes Supplementary Methods and Supplementary Figures.** Figure S1, the top gene expression network in males at <3 hours following cardioembolic stroke. Figure S2, cell-type specific gene expression in female and male following cardioembolic stroke. Figure S3, a network of interconnected significant pathways (Benjamini-Hochberg P<0.05) in females at <3 hours following cardioembolic stroke.
(PDF)

File S2 **Supplementary Tables.** Table S1, differentially expressed genes at 3 h, 5 h, and 24 h following cardioembolic stroke. Table S2, significantly enriched canonical pathways at 3 h, 5 h, and 24 h following cardioembolic stroke in male and female. Table S3, significantly enriched disease and function categories at 3 h following cardioembolic stroke in female and in male. Table S4, significantly enriched upstream regulators at 3 h following cardioembolic stroke in female and in male.
(XLSX)
