# Supplementary material for: Gene Expression in Peripheral Immune Cells following Cardioembolic Stroke Is Sexually Dimorphic
Source: PLoS One. 2014 Jul 18;9(7):e102550. doi: 10.1371/journal.pone.0102550 (PMC4103830; doi:10.1371/journal.pone.0102550)
Supplement: File S1 — Includes Supplementary Methods and Supplementary Figures. Figure S1, the top gene expression network in males at <3 hours following cardioembolic stroke. Figure S2, cell-type specific gene expression in female and male following cardioembolic stroke. Figure S3, a network of interconnected significant pathways (Benjamini-Hochberg P<0.05) in females at <3 hours following cardioembolic stroke. (PDF) [file pone.0102550.s001.pdf]

SUPPLEMENTARY MATERIALS AND METHODS

Study Participants

Cardioembolic (CE) Stoke Patients

Subjects with CE stroke (n=23, 11 female, 12 male; 3 timepoints each=69 samples), were recruited through the Clot Lysis: Evaluating Accelerated Resolution of IVH (CLEAR) trial. It is a multicenter, randomized, double-blind safety study of recombinant tissue-type plasminogen activator (rtPA) and eptifibatide[1] (NCT00250991 at Clinical-Trials.gov). The Institutional Review Board at each site approved the protocol and informed consent was obtained before study entry. Whole blood was collected at three time points from each subject with CE stroke. The first blood sample (at $\leq$3 hours) was drawn before any treatment. After treatment, two blood samples were drawn at 5 hours and 24 hours after the onset of the CE stroke. CE stroke was diagnosed by a stroke neurologist with access to all clinical and diagnostic tests including neurovascular imaging data as described in[2]. The diagnosis of CE stroke required identification of at least 1 source of cardiac embolus and exclusion of strokes with other etiologies. Atrial fibrillation was identified using electrocardiogram, echocardiogram, and/or 24–48-hour cardiac monitoring as described in[1, 2].

Vascular Risk Factor Controls (VRFC)

Subjects with at least one cardiovascular risk factor (hypertension, diabetes mellitus, hyperlipidemia) were recruited from the SAVVY (Sex, Age and Variation in Vascular functionalitY) study (n=23, 11 female, 12 male) as described in [3]. These subjects had no history of symptomatic vascular disease, as the exclusion criteria were past history of cardiovascular disease (including stroke, coronary artery disease, peripheral artery disease or deep vein thrombosis), BMI > 46kg/m^2, history of cancer, chronic infection, autoimmune disease or blood dyscrasias.

RNA and Array Processing

Whole venous blood was collected into PAXgene tubes (PreAnalytiX), which stabilize the RNA and protect against degradation and *in vitro* transcription. Whole blood contains different cell types. Thus, RNA was derived primarily from leukocytes–polymorphonuclear leukocytes (granulocytes - neutrophils, basophils, and eosinophils) and agranulocytes (lymphocytes, monocytes, and macrophages), as well as from immature red blood cells and immature platelets. Total RNA from each blood sample was hybridized on whole-genome Affymetrix Human U133 Plus 2.0 GeneChips (Affymetrix, Santa Clara, CA) per manufacturer's protocols. Raw expression values (probe level data) were imported into Partek software (Partek Inc, St Louis, MO). They were log-transformed and normalized using Robust Multichip Average and our previously reported internal gene normalization method[4].

Statistical Analysis

Gene expression was analyzed as a function of diagnosis, age, race, sex, scan batch and sex-by-diagnosis interaction using Analysis of Covariance (ANCOVA). Genes with false discovery rate (FDR)-corrected $P \leq 0.05$ (multiple comparison correction) and |fold change| ≥1.2

on the particular contrast from the interaction term were considered differentially expressed. An additional ANCOVA model was evaluated, where vascular risk factors (VRFs) were accounted for (atrial fibrillation, diabetes, hyperlipidemia, hypertension). Genes, significant for any of these VRFs ($p<0.005$) were excluded. Females with CE stroke were compared with female VRFC and males with CE stroke were compared with male VRFC to identify the immune response to CE stroke in each sex. Results were overlapped to identify sex-specific gene expression. This analytical approach decreases bias related to hormonal differences between the sexes. The analyses addressed changes of gene expression at each time point. All statistical analyses were performed using Partek Genomics Suite 6.

Cell-type specific transcripts.

Cell-type specific expression was determined based on comparing our gene lists to the Haem Atlas of cell-specific transcripts in differentiated human blood cells[5], Supplementary Table 5). We re-annotated the Illumina probesets using the latest annotation (www.Illumina.com). As described in the Haem Atlas, all cell types used in their study were >95% pure based on flow cytometry analysis and inspection by microspcopy.

Significance of gene list overlaps

A hypergeometric test[6] was performed to estimate the significance of overlaps of different gene lists (*phyper* function in R, http://R-project.org). This test was used to estimate significance of overlaps between each of our gene lists and each of the Haem Atlas genes with cell-type specific transcripts. The significant overlaps are presented in Figure 3 and Figure S1 with # symbol.

A two-sample t-test between percents was performed to determine whether there was a significant difference between the number of male and female cell-type specific genes following CE stroke. For example, for the neutrophil-specific genes we found at 3 hours following CE stroke, the calculations consider the % of male neutrophil-specific genes in our date, the size of the 3h male gene list, the % of female neutrophil-specific genes in our data, and the size of the 3h female gene list. The analyses were performed in Statistics Calculator StatPac (http://www.statpac.com/statistics-calculator/).

Functional Analyses (Canonical Pathway Over-representation) of the Identified genes

Pathway analysis was performed through the use oof IPA (Ingenuity® Systems, www.ingenuity.com). Canonical Pathway Analysis identified the pathways from the IPA library of canonical pathways that were most significant to each gene list. The significance of the association between the dataset and canonical pathways was assessed by calculating a Fischer's Exact Test, determining the probability that each Canonical Pathway assigned to that dataset is due to chance alone. A Benjamini-Hochberg corrected $p<0.05$ was considered to be statistically significant for over-representation of the molecules in a given pathway. Thus, over-represented pathways are the ones which have more focus molecules than expected by chance, after accounting for multiple comparisons for the multiple pathways tested.

IPA Downstream Effects Analyses

The Downstream Effects IPA analyses enable the visualization of biological trends in an experiment, by considering the direction of changes (up- or down-regulation) in our data and comparing them to Ingenuity® Knowledge Base to predict whether there is an increase or decrease in certain biological processes (functions). The goal was to identify biological functions

that are expected to be increased or decreased given the observed gene expression changes (up- or down-regulation) in our gene lists. The analysis is based on prior knowledge of expected causal effects between genes and biological functions stored in the Ingenuity® Knowledge Base. The analysis examines genes in each of our gene lists that are known to affect each biological function and compares their direction of change to what is expected from the literature. If the observed direction of change is mostly consistent with a particular activation state of a biological function ("increased" or "decreased"), then a prediction is made about that activation state. For each biological function a statistical quantity is computed, called the activation z-score. The activation z-score is used to infer likely activation states of biological functions based on comparison with a model that assigns random regulation directions. The predicted direction of change for each function is based on the regulation z-score. A |z-score| ≥ 2 is considered significant. A function/biological process is increased, when the z-score≥2, and decreased in the z-score≤2. This algorithm is designed to reduce the chance that random data will generate significant predictions.

REFERENCES

1. Pancioli AM, Broderick J, Brott T, Tomsick T, Khoury J, Bean J, del Zoppo G, Kleindorfer D, Woo D, Khatri P, Castaldo J, Frey J, Gebel J, Jr., Kasner S, Kidwell C, Kwiatkowski T, Libman R, Mackenzie R, Scott P, Starkman S, Thurman RJ. The combined approach to lysis utilizing eptifibatide and rt-pa in acute ischemic stroke: The clear stroke trial. *Stroke*. 2008;39:3268-3276

2. Jickling GC, Xu H, Stamova B, Ander BP, Zhan X, Tian Y, Liu D, Turner RJ, Mesias M, Verro P, Khoury J, Jauch EC, Pancioli A, Broderick JP, Sharp FR. Signatures of cardioembolic and large-vessel ischemic stroke. *Ann Neurol*. 2010;68:681-692

3. Stamova B, Xu H, Jickling G, Bushnell C, Tian Y, Ander BP, Zhan X, Liu D, Turner R, Adamczyk P, Khoury JC, Pancioli A, Jauch E, Broderick JP, Sharp FR. Gene expression profiling of blood for the prediction of ischemic stroke. *Stroke*. 2010;41:2171-2177

4. Stamova BS, Apperson M, Walker WL, Tian Y, Xu H, Adamczy P, Zhan X, Liu DZ, Ander BP, Liao IH, Gregg JP, Turner RJ, Jickling G, Lit L, Sharp FR. Identification and validation of suitable endogenous reference genes for gene expression studies in human peripheral blood. *BMC Med Genomics*. 2009;2:49

5. Watkins NA, Gusnanto A, de Bono B, De S, Miranda-Saavedra D, Hardie DL, Angenent WG, Attwood AP, Ellis PD, Erber W, Foad NS, Garner SF, Isacke CM, Jolley J, Koch K, Macaulay IC, Morley SL, Rendon A, Rice KM, Taylor N, Thijssen-Timmer DC, Tijssen MR, van der Schoot CE, Wernisch L, Winzer T, Dudbridge F, Buckley CD, Langford CF, Teichmann S, Gottgens B, Ouwehand WH. A haematlas: Characterizing gene expression in differentiated human blood cells. *Blood*. 2009;113:e1-9

6. Fury W, Batliwalla F, Gregersen PK, Li W. Overlapping probabilities of top ranking gene lists, hypergeometric distribution, and stringency of gene selection criterion. *Conf Proc IEEE Eng Med Biol Soc*. 2006;1:5531-5534

Figure S1.

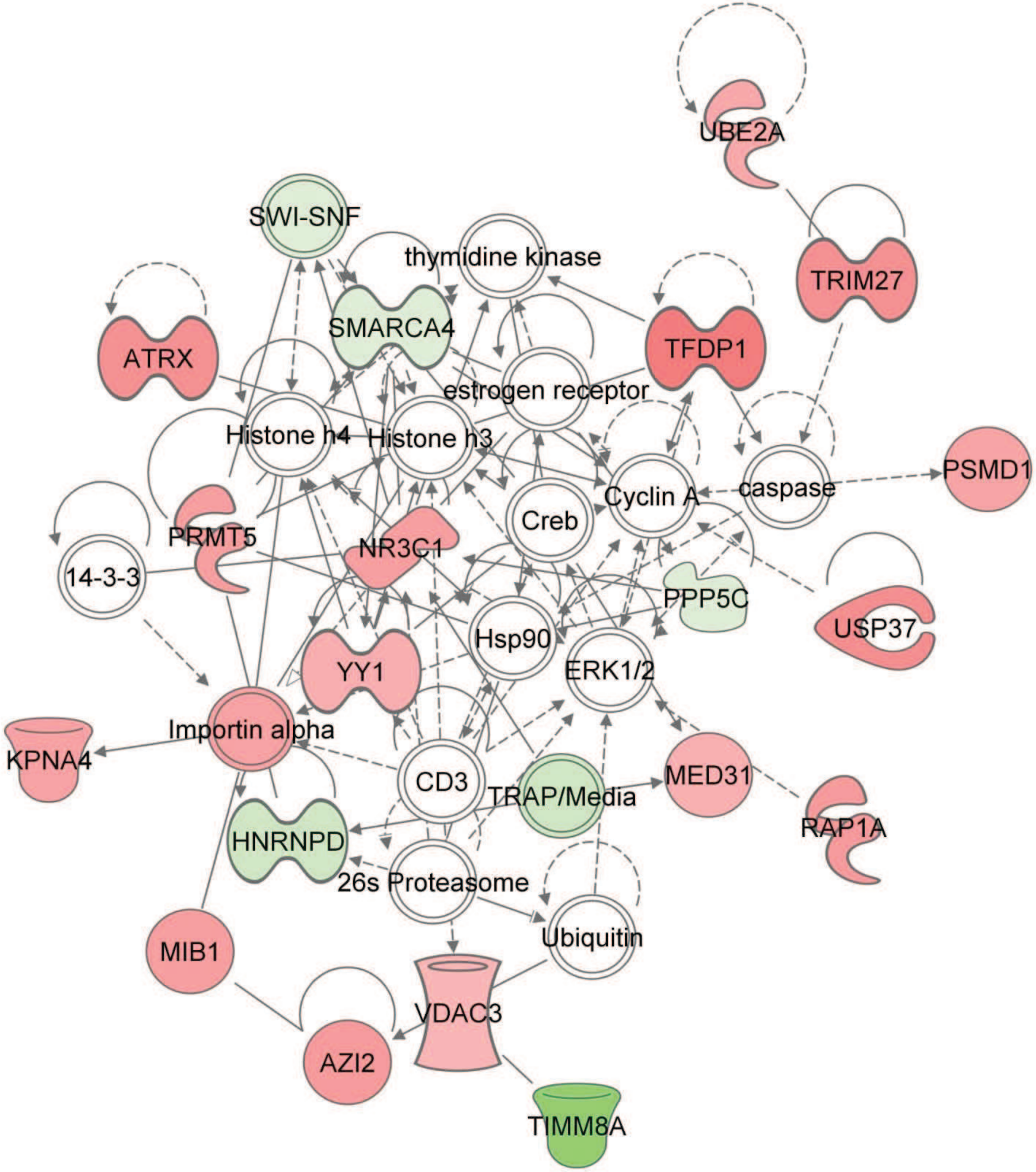

UBE2A
SWI-SNF
thymidine kinase
TRIM27
SMARCA4
ATRX
TFDP1
estrogen receptor
Histone h4
Histone h3
caspase
PSMD1
Cyclin A
Creb
PRMT5
NR3C1
14-3-3
PPP5C
USP37
Hsp90
YY1
ERK1/2
KPNA4
Importin alpha
CD3
TRAP/Media
MED31
RAP1A
HNRNPD
26s Proteasome
Ubiquitin
MIB1
VDAC3
AZI2
TIMM8A

Figure S2.

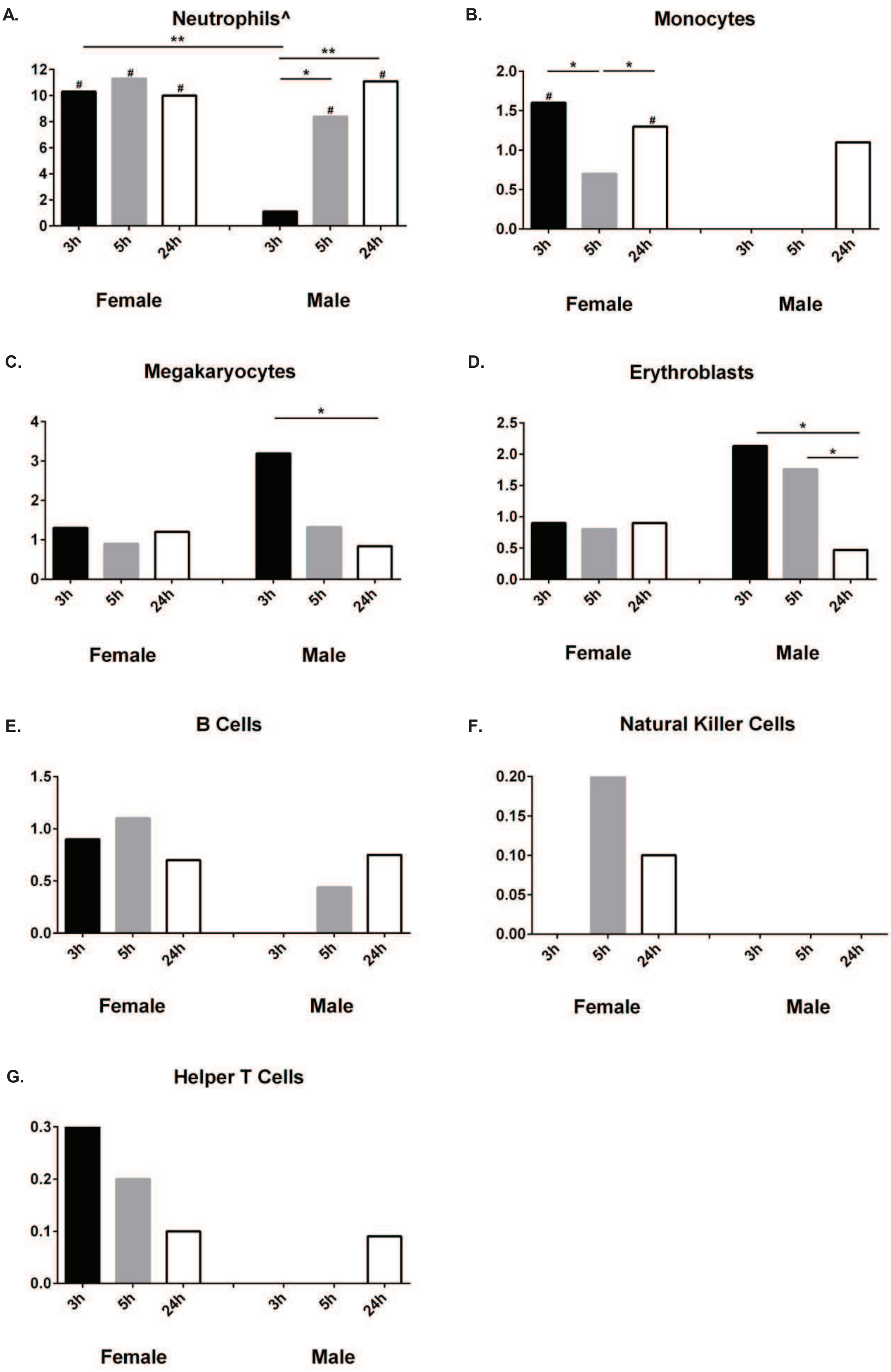

A.
Neutrophils^
**
**
*
#
12
10
8
6
4
2
0
3h
5h
24h
Female
Male
B.
Monocytes
*
*
#
2.0
1.5
1.0
0.5
0.0
C.
Megakaryocytes
*
4
3
2
1
0
D.
Erythroblasts
*
*
2.5
2.0
1.5
1.0
0.5
0.0
E.
B Cells
1.5
1.0
0.5
0.0
F.
Natural Killer Cells
0.20
0.15
0.10
0.05
0.00
G.
Helper T Cells
0.3
0.2
0.1
0.0

Figure S3.

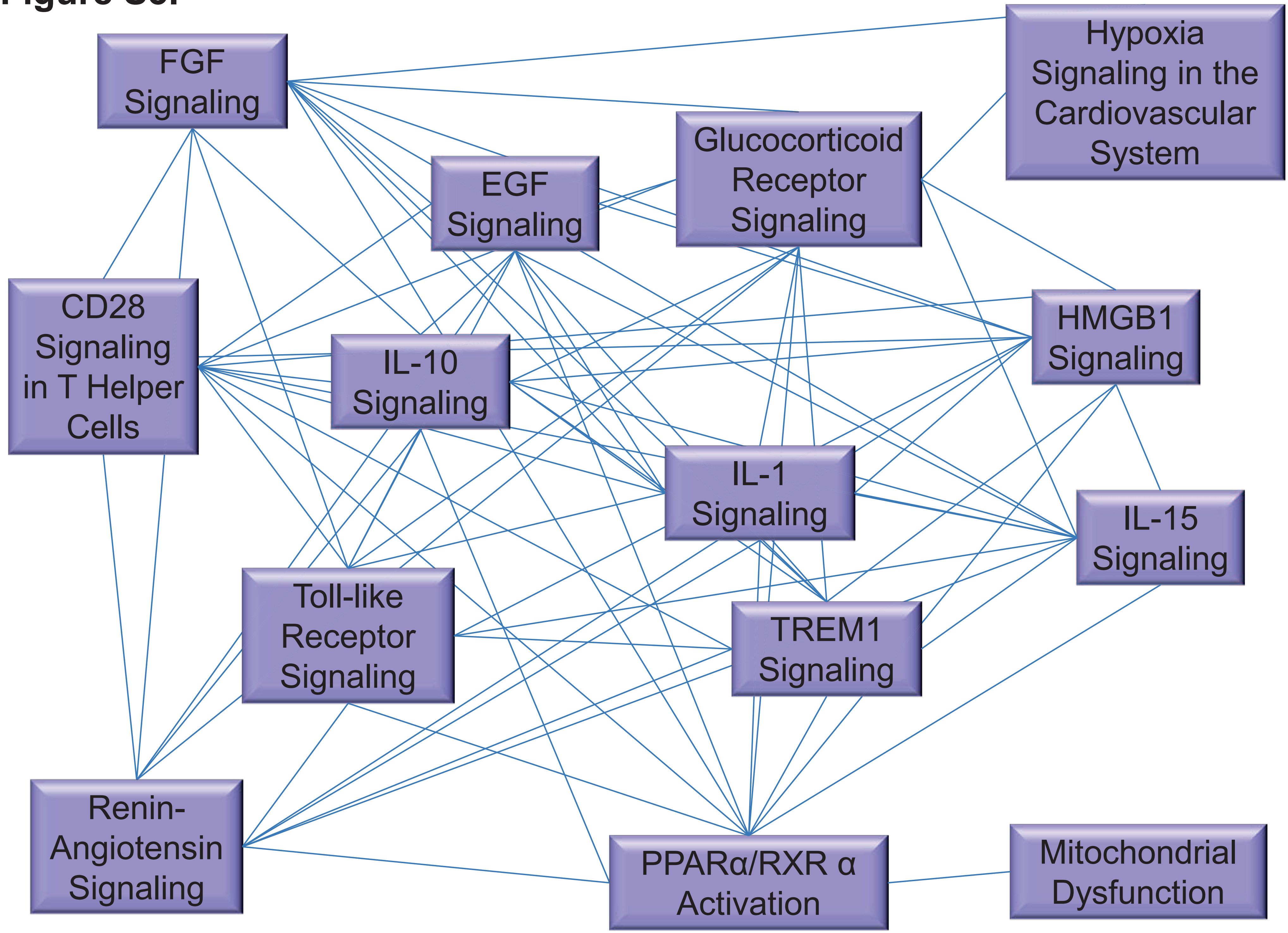
FGF Signaling
Hypoxia Signaling in the Cardiovascular System
Glucocorticoid Receptor Signaling
EGF Signaling
CD28 Signaling in T Helper Cells
IL-10 Signaling
HMGB1 Signaling
IL-1 Signaling
IL-15 Signaling
Toll-like Receptor Signaling
TREM1 Signaling
Renin-Angiotensin Signaling
PPARα/RXR α Activation
Mitochondrial Dysfunction

Supplementary Figures

Figure S1: The top gene expression network in males at <3 hours following cardioembolic stroke. Red – increased mRNA level; green – decreased mRNA level. Solid lines represent direct interactions, dashed lines – indirect interactions. The network was generate in Ingenuity (IPA) using filter considering experimentally observed relationships as well as data sources, such as BIND, BIOGRID, ClinicalTrials.gov, Cognia, DIP, Gene Ontology, GVK Bioscience, Ingenuity Expert Findings, Ingenuity ExpertAssist Findings, INTACT, Interactome, MINT, MIPS, Obesity Gene Map Database or TarBase.

Figure S2: As in Figure 3 with the addition of B-cell, Natural Killer cells, and T helper cells. Cell-type specific gene expression in female and male following cardioembolic stroke. Y-axis - % of genes from our gene list, which overlap with a cell-type specific gene list from Watkins et al, 2007. * $p<0.05$; ** $P<0.005$; # hypergeometric probability of overlap between our gene list and the cell-type specific gene list from Watkins et al, 2007 <0.05; ^ Watkins et al used positive selection, therefore granulocyte population consisted of three cell types (neutrophils, eosinophils, and basophils), all of which expressed CD66b and would have therefore been co-purified. However, the neutrophils are the largest percent.

Figure S3: A network of interconnected significant pathways (Benjamini-Hochberg $P<0.05$) in females at <3 hours following cardioembolic stroke.
